# MLN4924 suppresses neddylation and induces cell cycle arrest, senescence, and apoptosis in human osteosarcoma

**DOI:** 10.18632/oncotarget.9481

**Published:** 2016-05-19

**Authors:** Yi Zhang, Cheng-Cheng Shi, Hua-Peng Zhang, Gong-Quan Li, Shan-Shan Li

**Affiliations:** ^1^ Department of Orthopaedic Surgery, The First Affiliated Hospital of Zhengzhou University, Zhengzhou, Henan, China; ^2^ The Hormel Institute, University of Minnesota, Austin, MN, USA; ^3^ Department of Pharmacy, The First Affiliated Hospital of Zhengzhou University, Zhengzhou, Henan, China; ^4^ Department of Hepatobiliary and Pancreatic Surgery, The First Affiliated Hospital of Zhengzhou University, Zhengzhou, Henan, China; ^5^ Open and Key Laboratory of Hepatobiliary & Pancreatic Surgery and Digestive Organ Transplantation at Henan Universities, The First Affiliated Hospital of Zhengzhou University, Zhengzhou, Henan, China; ^6^ Department of Pathology, The First Affiliated Hospital of Zhengzhou University, Zhengzhou, Henan, China

**Keywords:** osteosarcoma, neddylation, MLN4924

## Abstract

Neddylation is a post-translational protein modification process associated with carcinogenesis and cancer development. MLN4924, a pharmaceutical neddylation inhibitor, induces potent anti-cancer effects in multiple types of cancers. In this study, we investigated the effects of MLN4924 on human osteosarcoma (OS). Levels of both NEDD8 activating enzyme E1 (NAE1) and ubiquitin-conjugating enzyme E2M (Ube2M), two critical components of the neddylation pathway, were much higher in OS tissues and cells than in normal osseous tissues and cells. MLN4924 treatment led to DNA damage, reduced cell viability, senescence and apoptosis in OS cells. Moreover, MLN4924 inhibited OS xenograft tumor growth in mice. Mechanistically, MLN4924 blocked the neddylation of cullins and induced accumulation of several tumor-suppressive substrates of Cullin-RING E3 ubiquitin ligases (CRLs), including CDT1, Wee1, p21, p27, Noxa, and p16. These results suggest clinical studies investigating the utility of MLN4924 for the treatment of OS are warranted.

## INTRODUCTION

Neddylation is a post-translational protein modification in which the ubiquitin-like protein NEDD8 is added to substrates by the sequential activity of the following enzymes: NEDD8-activating enzyme E1 (NAE1), NEDD8-conjugating enzyme E2, and substrate-specific NEDD8-E3 ligases [1–3]. Cullins, a group of proteins characterized by the presence of a distinct globular C-terminal domain (cullin-homology domain) and a series of N-terminal five-helix bundle repeats (cullin repeats), are well-known substrates of neddylation [4, 5]. Conjugation of NEDD8 induces conformational changes and confers cullins with the ability to recruit several other proteins and form Cullin-RING E 3 ubiquitin ligases (CRLs). In this way, neddylation promotes CRL-mediated ubiquitin/proteasome-dependent degradation of a number of proteins that are crucial for inhibiting cell growth and survival [5, 6]. Recent studies have suggested that upregulation of neddylation might contribute to tumorigenesis, unrestrained cell proliferation, and resistance to apoptosis in cancer cells. Accordingly, many studies have begun targeting the neddylation pathway as a therapy for cancer [7–9].

MLN4924 is a first-in-class pharmaceutical neddylation inhibitor discovered and developed by Millennium Pharmaceuticals as an anti-cancer drug [7]. NAE1 catalyzes the binding of MLN4924 to NEDD8 to form NEDD8-MLN4924 adducts, which block the neddylation of cullins and the subsequent disassembly of CRL complexes and inhibit E3 ubiquitin ligase activity [10, 11]. These events result in the accumulation of CRL substrates, which leads to DNA damage, cell cycle arrest, apoptosis, and senescence in cancer cells [7–12]. Preclinical studies of MLN4924 in hematological malignancies and solid tumors have demonstrated strong anti-cancer activity in multiple cancer types [13–23]. Clinical trials using MLN4924 as single agent or in combination with chemotherapy have been conducted for multiple kinds of human cancer [22–25]. Thus, MLN4924 may be beneficial for cancer patients who are resistant to conventional therapies.

Osteosarcoma (OS) is a rare malignant bone neoplasm which primarily affects children and young adults. Although the development of systemic chemotherapies over the past two decades has improved survival in patients with primary OS, there are still no effective treatments for OS patients with recurrences and metastases. Aberrant regulation of the ubiquitin/proteasome system (UPS) has been associated with the development and progression of osteosarcoma [26, 27]. However, it remains unclear whether the neddylation pathway is involved in UPS dysregulation in human OS and whether MLN4924 could be useful for managing OS.

## RESULTS

### The Neddylation pathway is activated in human osteosarcoma

**Figure 1:**
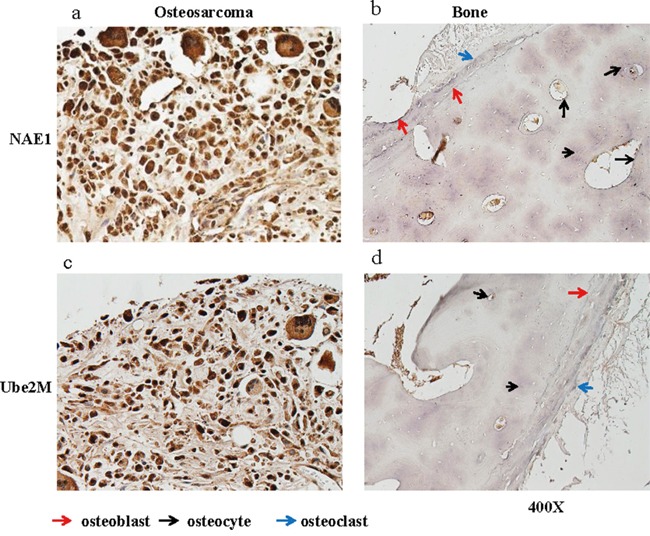
NAE1 and Ube2M levels are elevated in OS tumor tissues Levels of NAE1 and Ube2M in de-paraffinized sections of OS tumor tissues and of normal bone tissues were examined by immunohistological staining. Representative NAE1 staining in OS tumor tissue **a.** and in normal bone tissue **b.** are shown. Representative Ube2M staining in OS tumor tissue **c.** and in normal bone tissue **d.** are shown.

To evaluate whether the neddylation pathway is activated in OS, levels of the key components NAE1 and Ubiquitin-Conjugating Enzyme E2M (Ube2M) were examined by immunohistochemical (IHC) staining analysis in a tissue array containing tumor tissues from 40 OS patients (4 stage I and 36 stage II cases). Normal osseous tissues from healthy donors were used as controls. Staining intensity scores from 0 (weakest) to 3 (strongest) were assigned to each sample by three trained pathologists. Scores below 1 were considered low expression, and scores of 1 or higher were considered high expression. The neddylation enzymes were more highly expressed (score ≥ 1) in the majority of tumor tissues than in osseous cells in normal bone tissues. Specifically, 90% (36/40) of the OS tumor samples had high levels of NAE1, and 95% (38/40) had high levels of Ube2M (Figure 1a, 1c). In contrast, three types of osseous cells (osteocytes, osteoblasts, and osteoclasts) in normal bone tissues weakly expressed both NAE1 and Ube2M (Figure 1b, 1d). To complement the IHC findings, we examined levels of several key components of the neddylation pathway (SJSA-1, Saos-2, MG-63, and HOS) in 4 OS cell lines as well as in normal human osteoblasts (NHOst). The OS cell lines and NHOsts had comparable levels of NEDD8. However, all 4 OS cell lines had much higher levels of NAE1 and Ube2M than NHOsts. The OS cell lines and NHOsts also had comparable Cullin1 levels. However, the OS cell lines had much higher levels of neddylated Cullin1, indicating that the neddylation pathway is overactive in OS cells (Supplementary Figure S1).

To investigate whether OS cell survival depends on activation of the neddylation pathway, we knocked down expression of the key molecule NAE1 using two siRNA oligos in SJSA-1 cells. Inhibition of NAE1 reduced cell viability (Supplementary Figure S2). This result suggests that the neddylation pathway is essential for OS cell survival. Collectively, these findings suggest that the neddylation pathway is broadly activated in human OS and that neddylation might be an effective target for OS molecular therapy.

### MLN4924 inhibits cell viability in human OS cells

**Figure 2:**
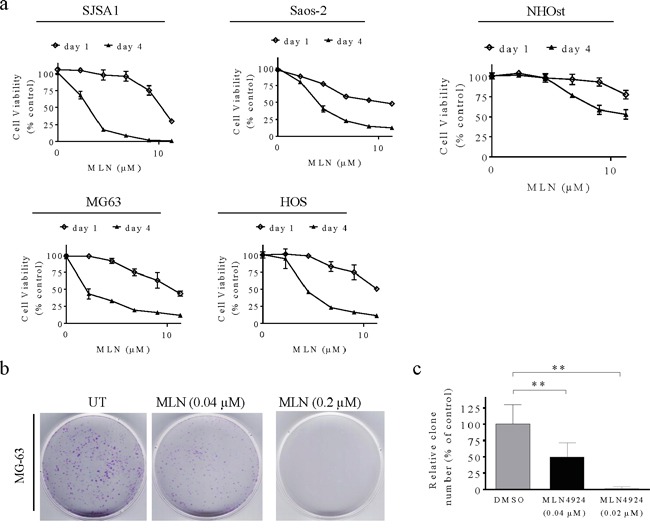
MLN4924 reduces human OS cell viability **a.** Human OS cell lines and normal osteoblasts were treated with serial dilutions of MLN4924 for 1 or 4 days and cell viability was determined using MTT assays. Representative inhibitory curves from three independent experiments are shown for each cell line. **b**, **c.** MG-63 cells were seeded into 60 mm × 15 mm petri dishes at 3,000 cells per dish in triplicate and treated with MLN4924 for 12 days, followed by 0.01% (w/v) crystal violet staining and colony counting. (b) Representative images of three independent experiments are shown for colony formation. (c) Graph of the relative number of colonies formed (the results of three independent experiments, expressed as mean ± SEM). (**p<0.01).

We next evaluated the ability of MLN4924 to reduce cell viability *in vitro* by treating 4 human OS cell lines and NHOsts with serial dilutions of the drug (0.008–25 μM) for 1 and 4 days. One day of treatment with 10 μM MLN4924 only reduced cell viability by 10-30% in the 4 OS cell lines. However, 4 days of MLN4924 treatment strongly and dose-dependently reduced cell viability in all 4 OS cell lines (Figure 2a). The IC50 (the concentration that inhibits cell growth by 50%) values of MLN4924 were 0.073, 0.071, 0.19, and 0.25 μM for the SJSA-1, MG-63, Saos-2, and HOS cell lines, respectively (Figure 2a). Notably, 5 μM MLN4924 almost completely inhibited cell viability in SJSA-1 and MG-63 cells, indicating a strong cytotoxic effect of MLN4924 in these two OS cell lines. We therefore used these two cell lines for further investigations. In contrast, 4 days of 10 μM MLN4924 only inhibited NHOst cell growth by about 50%.

We performed clonogenic assays to determine the long-term anti-proliferative effects of MLN4924 in MG-63 and SJSA-1 cells. Twelve days of MLN4924 treatment strongly inhibited clone formation in a dose-dependent manner in both OS cell lines (Figure 2b, 2c, Supplementary Figure S3). Specifically, 0.04 μM MLN4924 reduced clone numbers, and 0.2 μM MLN4924 completely blocked clone formation. Together, these findings demonstrate that inhibiting the neddylation pathway with MLN4924 effectively reduces survival and growth of OS cells.

### MLN4924 inhibits neddylation of cullins and blocks the degradation of CRL substrates

**Figure 3:**
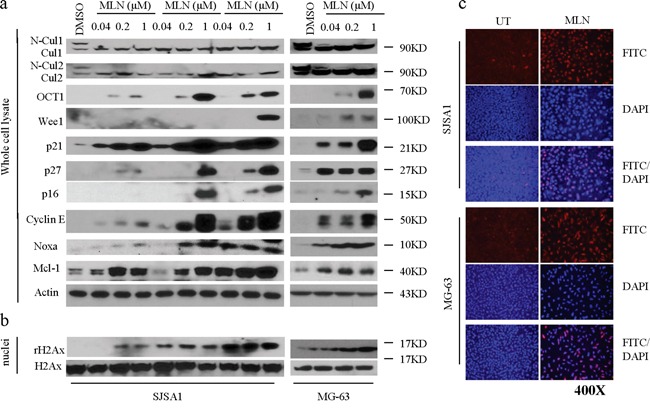
MLN4924 inhibits neddylation of cullins and induces accumulation of CRL substrates in OS cells **a.** SJSA-1 and MG-63 OS cells were treated with increasing concentrations of MLN4924 (0.04, 0.2, and 1 μM) as indicated. Levels of cullin1, cullin2, and a panel of CRL substrates were examined by western blotting analysis in the whole cell lysates. Actin was used a loading control. **b.** Treated cells were cell fractionated and levels of rH2Ax and H2Ax were examined by western blotting analysis in the nuclei of cell lysates. **c.** OS cell lines were treated with MLN4924 at 1 μM for 48 h. γ-H2AX staining was detected by immunofluorescence.

To determine the mechanisms underlying MLN4924-induced inhibition, we treated SJSA-1 cells with three sub-toxic concentrations of MLN4924 (0.04, 0.2, or 1 μM) for 6, 24, or 48 h and examined the effects on neddylation of cullins and levels of tumor-suppressive CRLs substrates using western blotting analysis. MLN4924 treatment rapidly blocked neddylation of cullin 1 and cullin 2, two tumor-associated cullin family members (Figure 3a). This inhibitory effect indicated that CRL E3 ligase function and the degradation of CRLs substrates were also inhibited. Indeed, MLN4924 treatment led to rapid accumulation of a panel of CRLs substrates that are associated with cell proliferation and apoptosis. These proteins included CDT1, p27, p21, Wee1, Noxa, p16, and cyclin E (Figure 3a). Importantly, MLN4924 increased p21 levels very rapidly. p21 levels peaked within 6 h of MLN4924 treatment and were sustained for 48 h in the presence of MLN4924 (Figure 3a). This demonstrated that MLN4924 increased rapid turnover of p21. Similar effects of MLN4924 on tumor-associated CRLs substrates were also observed in the MG-63 cells. These results indicated that inhibition of cullin neddylation by MLN4924 causes accumulation of tumor-suppressive proteins in OS cells.

### MLN4924 induces DNA damage in OS cells

MLN4924 strongly increased levels of the DNA replication licensing protein CDT1 in OS cells, which might cause DNA damage. We thus examined levels of γ-H2AX (phosphorylated H2AX), a typical biomarker of DNA damage, in OS cells after MLN4924 treatment. Cell fractionation and western blotting analysis showed that MLN4924 treatment markedly increased γ-H2AX levels in the nuclei of both OS cell lines as compared to DMSO (vehicle) treatment (Figure 3b). We next performed immunofluorescence staining to visualize MLN4924-induced DNA damage in OS cells. 24 h of MLN4924 treatment substantially increased γ-H2AX fluorescence in the nuclei of both OS cell lines as compared to DMSO treatment (Figure 3c). Quantitative analysis showed that 1 μM MLN4924 resulted in γ-H2AX fluorescence signals in 90-100% of OS cells, while the DMSO treatment resulted in this signal in only 5% of cells. These data collectively showed that MLN4924 triggered severe DNA damage in OS cells.

### MLN4924 induces G2 cell-cycle arrest and senescence in OS cells

**Figure 4:**
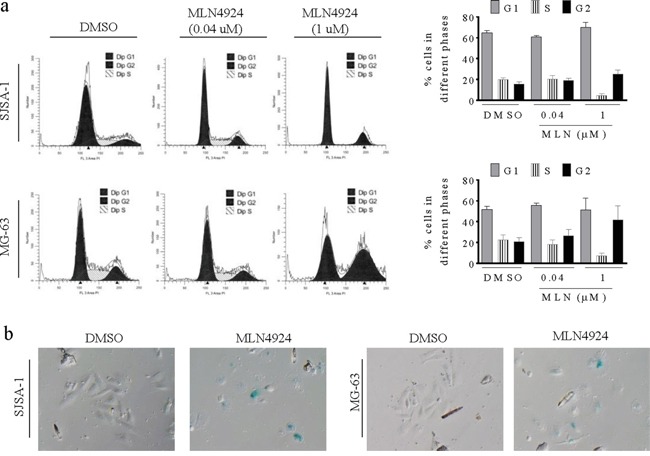
MLN4924 arrests cell cycle in the G2 phase and induces senescence in OS cell lines **a.** SJSA-1 and MG-63 OS cells were treated with MLN4924 for 48 h, stained with PI, and examined with flow cytometry assays. (left panels) Representative plots of the cell cycle phases from three independent experiments are shown for SJSA-1 and MG-63 cells treated with 0.04 μM or 1 μM MLN4924. (right panels) The fractions of SJSA-1 an MG-63 cells in each phase of the cell cycle are shown. **b.** SJSA-1 and MG-63 OS cells were treated with 1 μM MLN4924 for 48 h and senescence was examined by β-galactosidase staining.

Since MLN4924 increased the levels of several critical cell cycle regulators, including Wee1, p21, p27, and p16, and induced DNA damage in OS cells, we next examined whether these alterations resulted in cell cycle arrest and senescence in OS cells. In flow cytometry cell cycle analysis using propidium iodide DNA staining, a low concentration of MLN4924 (0.04 uM) modestly affected the cell cycle distribution, and a high concentration (1 μM) increased the population of cells in the G2 phase while reducing the S phase population (Figure 4a).

Furthermore, we observed that a fraction of cells became flat and greatly enlarged after MLN4924 treatment, suggesting an increase in cell senescence. We performed a β-galactosidase staining assay and found that most MLN4924-treated OS cells were stained (Figure 4b), confirming MLN4924-induced senescence, which is a common response to the upregulation of p16, p21, and p27, and to DNA damage.

### MLN4924 triggers apoptosis in OS cells

**Figure 5:**
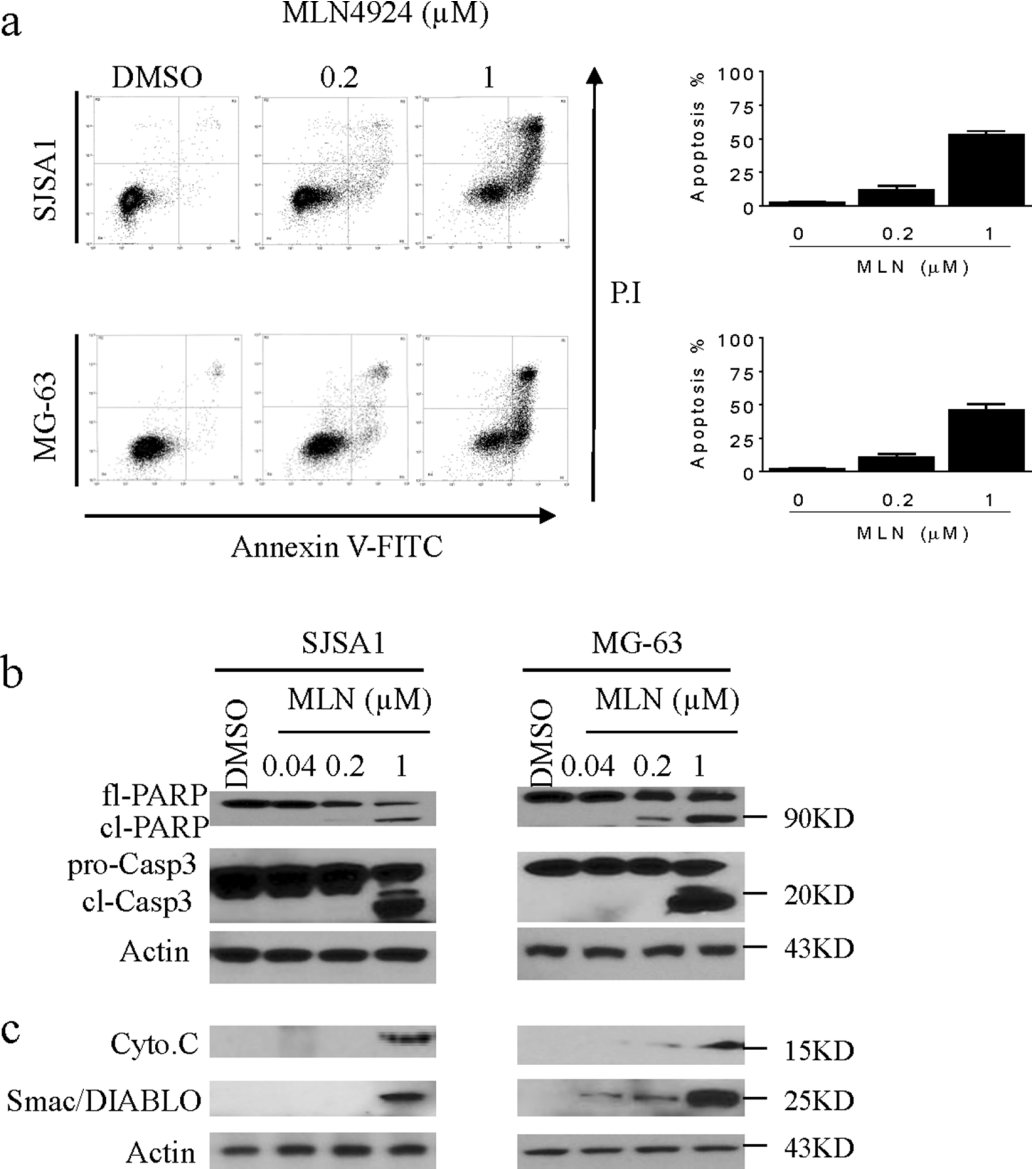
MLN4924 induces apoptosis in OS cell lines **a.** SJSA-1 and MG-63 OS cells were treated with MLN4924 for 48 h, stained with Annexin-V-FITC and PI, and examined with flow cytometry assays. (left panels) Representative plots of apoptotic cell distributions from three independent experiments are shown for SJSA-1 and MG-63 cells treated with MLN4924. (right panels) The percentages of SJSA-1 and MG-63 cells in apoptosis are shown. **b.** SJSA-1 and MG-63 cells were treated with MLN4924 for 48 h, and levels of full-length PARP (fl-PARP), cleaved PARP (cl-PARP), pro-caspase-3 (pro-C3), and cleaved-caspase-3 (cl-C3) were analyzed by western blotting in the whole cell lysates. Actin was used as a loading control. **c.** Treated cells were fractionated; levels of cytochrome c (cyto.c) and Smac/Diablo were examined in the cytosolic fraction by western blotting. Actin was used as a loading control.

Because MLN4924 induced accumulation of the pro-apoptotic protein Noxa, we investigated the apoptotic effect of MLN4924 in OS cell lines. MLN4924 at 1 μM for 24 h induced morphological changes that are highly characteristic of apoptosis, such as shrinkage, rounding, and floating, in both OS cell lines. These morphological changes became even more prominent after treatment for longer periods of time. These observations indicated that MLN4924 triggered apoptosis in OS cells. We then used Annexin V-FITC/PI-labeling flow cytometry to confirm this apoptotic effect. As shown in Figure 5a, treatment with MLN4924 at 0.2 μM or 1 μM for 48 h induced apoptosis in 8% and 53% of SJSA-1 cells, respectively; the same treatments induced apoptosis in 11% and 46% of MG-63 cells, respectively (Figure 5a). We also measured levels of apoptosis-associated proteins in cell lysates using cell fractionation and western blotting analysis. MLN4924 activated apoptosis signaling as evidenced by the activation of caspase-3, cleavage of PARP, and the release of mitochondrial pro-apoptotic proteins cytochrome c and Smac into the cytosol (Figure 5b, 5c).

The role of Noxa in MLN4924-mediated anti-OS activity was investigated using specific siRNA against Noxa. Suppression of Noxa inhibited MLN4924-induced cell death. Although the effect was not as strong as that observed previously in other cancers, these results still suggest an important pro-apoptotic role for Noxa in MLN4924-mediated anticancer activity (Supplementary Figure S4).

### MLN4924 suppresses growth of OS xenografts *in vivo* by inhibiting proliferation and inducting apoptosis in tumor cells

**Figure 6:**
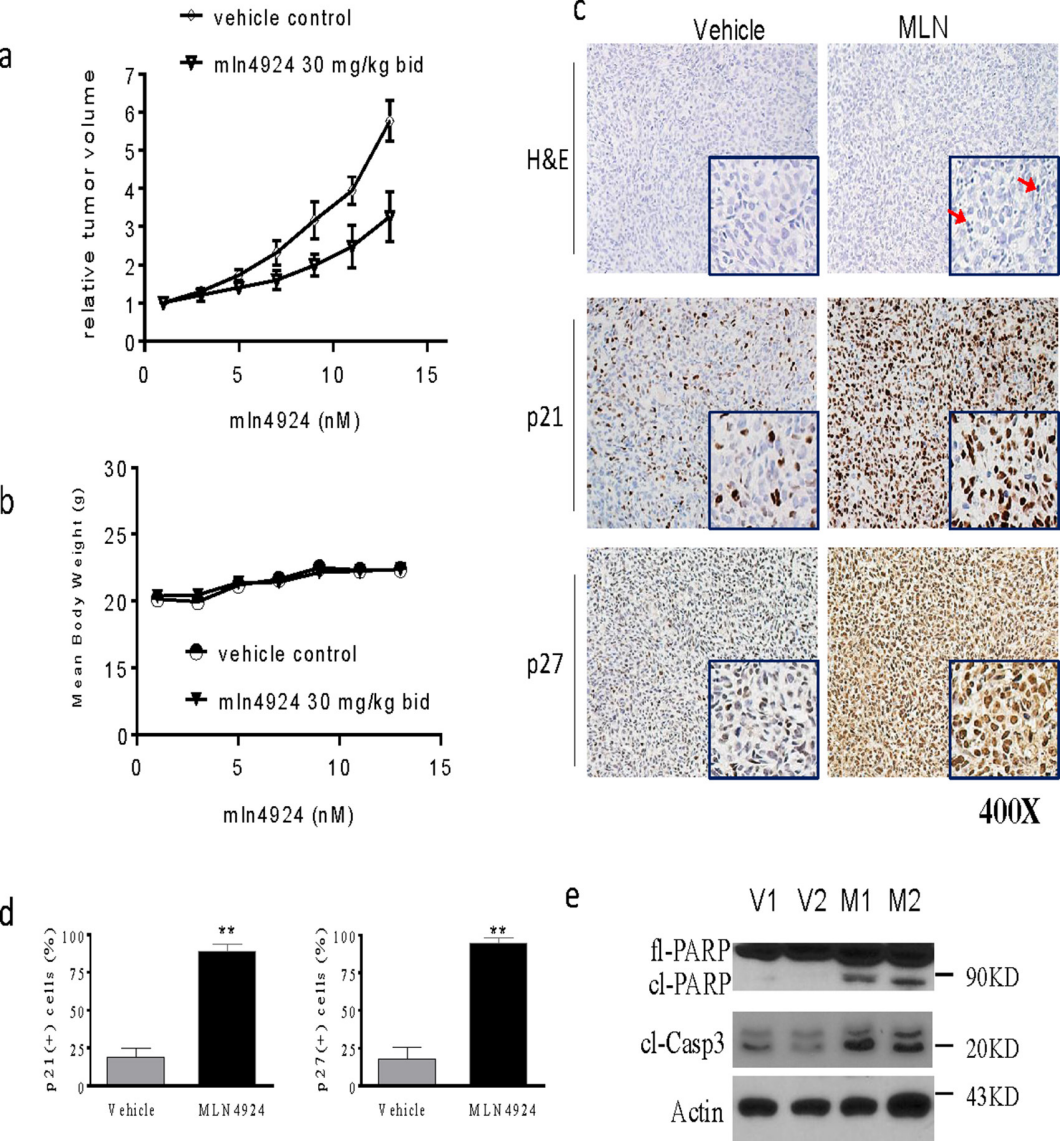
MLN4924 inhibits tumor growth in subcutaneous OS models SJSA1 cells were implanted subcutaneously (s.c.) in mice and allowed to grow until tumors reached a size of approximately 120 mm^3^. Xenografted mice were randomized into two groups and treated twice daily (bid) with 30 mg/kg MLN4924 or vehicle 5 times a week for 2 weeks. Tumor volume and body weight were monitored during the treatments. **a.** The graph shows changes in tumor volumes. The data are shown as mean ± SE (n = 8). Tumor volumes from the two groups were analyzed using a one-way ANOVA (*p < 0.05). **b.** The graph shows body weight of mice during treatment. MLN4924 had no effect on body weights. **c, d.** Histological alterations in OS xenograft tissues were examined by H&E staining (top panels). Levels of p21 and p27 in OS xenograft tissues were examined by IHC analyses. (c) Representative images are shown for vehicle and treated tumors, and (d) the percentages of p21- and p27-positive cells in tumor tissues were quantified and plotted in the graphs. **e.** Levels of fl-PARP, cl-PARP, and cl-C3 in tissue lysates were examined with western blotting analysis. Actin was used as a loading control.

To investigate the anti-tumor effects of MLN4924 *in vivo*, we employed OS xenografts in mice. SJSA-1 cells were injected subcutaneously (s.c.) into the flanks of nude mice; after palpable xenograft tumors were established, mice were randomly assigned into treatment or control groups. MLN4924 was injected s.c. into 8 mice twice daily at 30 mg/kg body weight for a total of 14 days. A cohort of 8 mice harboring xenograft tumors was injected with vehicle alone as a control. Suppression of tumor growth was evident beginning on the fifth day of treatment with MLN4924. The average tumor volume in the MLN4924-treated group was smaller than that in the vehicle group on day 14. Moreover, MLN4924 treatment delayed OS xenograft growth (*p* < 0.05; Figure 6a). The mice treated with MLN4924 showed no obvious signs of toxicity (based on body weight, food and water intake, activity, and general examination) during treatment (Figure 6b).

To elucidate the mechanisms underlying MLN4924-induced anti-OS activity, we performed histochemical studies and immunostaining of formalin-fixed paraffin-embedded tumor sections. Histological analysis of H&E staining revealed the presence of tissue destruction and condensation of nuclei (red arrows) in OS tissues obtained from mice treated with MLN4924 (Figure 6c, upper panels). IHC analysis revealed that almost all OS tumor cells were positive for p21 and p27 after MLN4924 treatment (Figure 6c, middle and lower panel, Figure 6d). Western blotting analysis showed that MLN4924 treatment increased PARP cleavage and caspase-3 activation in OS tissues (Figure 6e). Taken together, these experiments indicated that MLN4924 suppressed growth and induced apoptosis in xenograft OS tumors in mice.

## DISCUSSION

OS is highly malignant bone tumor which affects young people, usually occurring during the adolescent growth period. When treated appropriately, patients with low grade cancer have a good prognosis; however, there is no treatment for those whose disease has spread beyond the primary tumor site or who respond poorly to conventional therapies [26, 27]. Novel treatment strategies for OS patients are therefore needed. MLN4924 is a neddylation inhibitor with potent anti-proliferative and apoptotic effects in numerous cancers. Owing to its excellent pharmacodynamic and pharmacokinetic properties, as well as its low toxicity, this promising novel anticancer drug is currently under clinical trials in human cancer [7, 25]. In this study, we investigated whether MLN4924 could be therapeutically beneficial for human OS.

We first demonstrated that the neddylation pathway is activated in most OS cells, as evidenced by comparative analysis of NAE1 and Ube2M levels in OS tumor tissues and in healthly bone tissues. Over 90% of OS tumor tissues had high levels of these two critical neddylation pathway components, while levels were much lower in osseous cells from normal bone tissues. Moreover, inhibition of the neddylation pathway by knockdown of NAE1 reduced the viability of SJSA-1 cells. Together, these findings imply that neddylation plays an essential role in sustaining OS cell survival and suggest that MLN4924-induced inhibition of neddylation might help manage OS in most patients. We further showed that MLN4924 reduced proliferation and increased apoptosis in cultured OS cells, and also strongly inhibited clonogenic survival and growth. Moreover, using a mouse OS xenograft tumor model, we showed that 2 weeks of MLN4924 treatment inhibited tumor growth by suppressing proliferation and inducing apoptosis without causing severe toxicity. Jointly, these findings provide compelling evidence that MLN4924 may be a promising treatment for human OS.

Analyses of the underlying mechanisms revealed that MLN4924 inhibits cell proliferation by inducing cell cycle arrest and senescence in OS cells. Moreover, western blotting analysis showed that MLN4924 treatment increased levels of several critical cell cycle regulators, including cyclin-dependent kinase inhibitors p21, p27, and p16, G2 cell cycle checkpoint Wee1, and the DNA replication factor CDT1, in OS cells. Therefore, our data support the conclusions of previous studies in other cancers that MLN4924 inhibits cell proliferation via multiple pathways [7-11, 28-30].

Several lines of evidence from this study support the essential role of apoptosis in MLN4924-mediated anti-OS activity. First, MLN4924 treatment lead to typical apoptotic morphological changes in OS cells. Second, MLN4924 treatment increased the number of Annexin-V-FITC-positive OS cells. Finally, MLN4924 treatment activated apoptosis signaling, including the cleavage of caspases and PARP and the release of pro-apoptotic mitochondrial proteins cytochrome c and Smac/DIABLO into the cytoplasm.

The Bcl-2 family members promote apoptotic signaling [31–33]. Previous studies have attributed MLN4924-induced apoptosis to the accumulation of the pro-apoptotic BH3-only protein Noxa in other cancers [34, 35]. We therefore investigated the role of Noxa in MLN4924-medaited anti-OS activity. MLN4924 treatment markedly increased Noxa levels in OS cells, and suppression of Noxa by siRNA reduced cell death. Thus, Noxa is important for MLN4924-induced apoptosis.

Intriguingly, we noted an apparent discrepancy between the ability of MLN4924 to inhibit neddylation of cullins and to strongly suppress cell growth and induce apoptosis in OS cells. For example, even a very low concentration of MLN4924 (0.04 μM) completely inhibited the neddylation of cullins, but a higher concentration (0.2 μM) was needed to strongly inhibit cell proliferation, and only a much higher concentration (1 μM) strongly induced apoptosis in OS cells. Moreover, although MLN4924 has broad-spectrum anticancer efficacy in a range of cancer types, it only modestly inhibits tumor growth and is not able to induce tumor regression in most tumor types [7, 29, 30]. For example, we observed here that 30 mg/kg MLN4924 twice daily (half of the maximum tolerable dose) only inhibited tumor growth by about 50%. This study and several previous studies show that, in addition to inducing accumulation of tumor-suppressive proteins, MLN4924 also dramatically increases levels of several pro-survival oncogenic proteins, such as cyclin E and Mcl-1 [17, 29, 30, 34]. The MLN4924-induced accumulation of these pro-survival proteins may reduce its anticancer activity, and combined treatment with other antitumor agents that suppress these proteins may further improve the therapeutic efficacy of MLN4924.

## MATERIALS AND METHODS

### Cell lines and compound preparation

The OS cell lines SJSA-1, MG-63, Saos-2, and HOS were purchased from the China Center for Type Culture Collection (Wuhan, China) and maintained in PRMI1640 (HyClone/Thermo Fisher Scientific, Beijing, China) supplemented with 10% heat-inactivated fetal bovine serum (Hangzhou Sijiqing Biological Engineering Materials Co., Ltd, Hangzhou, China). Normal Human Osteoblasts (NHOst) were purchased from Lonza (Shanghai, China) and were cultured in Osteoblast Growth Media (Lonza). MLN4924 was purchased from Selleck Shanghai (Shanghai, China). MLN4924 was dissolved in Dimethyl sulfoxide (DMSO) at a stock concentration of 10 mmol/L and stored at −20°C.

### MTT cell viability assay

Cell viability was measured using a 3-[4,5-dimethylthiazol-2-thiazolyl]-2,5-diphenyl-tetrazolium bromide (MTT) assay that is based on mitochondrial conversion of MTT from a soluble tetrazolium salt into an insoluble colored formazan precipitate, which was dissolved in DMSO and quantified by spectrophotometry (Thermo Multiskan MK3; Thermo Labsystems, Shanghai, China) to obtain optical density (OD) values. OS cells were plated in 96-well culture dishes (Costar, Cambridge, MA, USA) at a density of 1000–2000 cells/well in 100 μL of medium. Serial dilutions were generated from a stock solution of MLN4924 to the desired concentrations. All experimental concentrations were replicated in triplicate. Four hours before the desired time points, 10 μL of 10 mg/mL MTT was added. After a 4h incubation, all media was removed from wells, and 100 μL of DMSO was added. Absorbance percentages for treated cells relative to those of untreated control samples were plotted as a function of drug concentration (log scale). Inhibition of cell viability was measured by the percentage of viable cells relative to the control: % inhibition = 100% × ODT / ODC, where ODT is the average OD value of the treated samples and ODC is the average OD value of the control samples.

### Cell death, flow cytometry, and clonogenic assays

Cell death was quantified by microscopic examination in trypan blue exclusion assays. Cell cycle analysis was conducted using propidium iodide (PI, 50 μg/mL in PBS) and flow cytometry with a BD LSR II system (BD Biosciences, Shanghai, China). The apoptosis assay was performed by staining cells with Annexin-V-FITC/PI and examining apoptosis by flow cytometry with a BD LSR II system (BD Biosciences, Shanghai, China). For clonogenic assays, 3,000 cells were seeded into 60 mm × 15 mm petri-dishes in 5 mL of medium, treated as indicated, and maintained for 12 days at 37°C in a 5% CO2 incubator. Cells were then washed with drug-free medium, stained with 0.01% (w/v) crystal violet, and cell colonies (> 50 cells) were counted. The assays were performed in duplicate with at least three replications per treatment.

### Cell fractionation

Histone protein isolation was performed with a commercial kit from Sigma (9064-47-5, Beijing, China) according to the manufacturer's protocol. Cytosolic cell lysate was isolated manually. Briefly, OS cells were treated as indicated, collected, washed with PBS, and suspended in 5 volumes of chilled buffer A (250 mM sucrose, 20 mM HEPES, 10 mM KCl, 1.5 mM MgCl2, 1 mM EDTA, 1 mM EGTA, 1 mM DL-dithiothreitol [DTT], 17 μg/mL phenylmethylsulfonyl fluoride [PMSF], 8 μg/mL aprotinin, and 2 μg/mL leupeptin [pH 7.4]) on ice for 15 min. Cells were homogenized using an ice-cold cylinder cell homogenizer (20–25 strokes). Homogenized cell lysates were separated by centrifugation at 750 g for 10 min, and the supernatants were further centrifuged at 10,000 g for 20 min. The remaining supernatant was used as the cytosolic fraction and subjected to western blot analysis.

### Western blotting

Western blotting was performed as described previously [36–38]. Cells were lysed using radioimmunoprecipitation (RIPA) assay lysis buffer (PBS containing 1% NP40, 0.5% Na-deoxycholate, and 0.1% SDS) supplemented with 1 μmol/L phenylmethylsulfonyl fluoride and 1 protease inhibitor cocktail tablet per 10 mL on ice for 20 min, and lysate protein concentrations were determined using the Bio-Rad protein assay kit according to the manufacturer's instructions. Proteins were electrophoresed onto 4–20% SDS-PAGE gels and transferred onto polyvinylidene difluoride membranes. Following blocking in 5% milk, the membranes were incubated with a specific primary antibody, washed, and incubated with horseradish peroxidase–linked secondary antibody (GE Healthcare, Beijing, China). Signals were visualized with chemiluminescent horseradish peroxidase antibody detection reagent (Denville Scientific, Guangzhou, China).

The antibodies against Cul-1 (H-213) (sc-11384), Cul-2 (H-300) (sc-10781), Caspase-9 (96.1.23) (sc-56076), Caspase-3 (H-277) (sc-7148), Mcl-1 (S-19) (sc-819), Wee1 (C-20) (sc-325), Oct-1 (C-21) (sc-232), p-Histone H2AX (9F3) (sc-56743), H2A.X Antibody (M-20) (sc-54607), Cyclin E (C-19) (sc-198), Noxa (114C307) (sc-56169), and Actin (C4) (sc-47778) were purchased from Santa Cruz Biotechnology (Shanghai, China). The antibodies against p27 (2552), p21 (2947), p16 (4824), Noxa (14766), cytochrome c (4280), Smac (15108), NAE1 (14321), and Ube2M (5641) were from Cell Signaling Technology (Shanghai, China).

### RNA interference

siRNAs oligos against Noxa and NAE1 were purchased from GE Dharmacon (Shanghai, China). Non-targeting control siRNAs (siNTC) were purchased from Qiagen (Shanghai, China). The siRNA transfections (30 pmol/L) were performed using Lipofectamine RNAiMax transfection reagent (Invitrogen, Shanghai, China).

### Immunohistochemistry (IHC)

A tissue microarray of 40 OS tissue sections and normal bone tissue sections was purchased from Alenabio Biotechnology (Xi'an, China). Tumor tissues were obtained from tumor-bearing mice treated with MLN4924 or vehicle control for 3 days. The antibodies used for IHC, p27 (2552), and p21 (2947) were purchased from Cell Signaling Technology (CST, Shanghai, China). IHC was performed following a standard protocol. Briefly, the sections were de-paraffinized by xylene, rehydrated in graded concentrations of ethanol, and boiled in antigen retrieval buffer (Abcam, Shanghai, China) in a microwave oven for 5 min. Slides were incubated with diluted antibodies at room temperature for 2 h. After incubation, the slides were washed three times with PBS, incubated with horseradish peroxidase (HRP)-conjugated antibody (Invitrogen, Shanghai, USA) at room temperature for 30 min, followed by incubation with ABC (avidin-biotin complex, Vectorlabs, Shanghai, China) for 30 min and visualization by the addition of 3,3′-diaminobenzidine tetrahydrochloride (DAB) reagent (Dako Diagnostics (Shanghai) Co., Ltd.), with hematoxylin as the counter stain. Images of stained slides were captured using a standard light microscope.

### Immunofluorescence

Cells were seeded into 8-chamber culture slides (BD Falcon), fixed with 4% formaldehyde at room temperature, and then rinsed with PBS. Cells were permeabilized with 0.5% Triton X-100 and 0.2% BSA in PBS for 10 minutes on ice and then blocked with 1% BSA in TBS-T for 1 hour at room temperature, followed by incubation with phospho-Histone H2A.X (Ser139) (1:500; CST) antibodyin 3% BSA in TBS-T for 2 hours at room temperature. The cells were then washed with TBS-T and incubated with Alkaline Phosphatase (AP) secondary antibody (1:1,000; VectorLab, Shanghai, China) at room temperature for 1 h. The cells were washed with TBS-T, stained with VectorRed, and coverslipped with ProLong with DAPI (Life Technologies). Images of stained slides were captured using a standard light microscope.

### Animal studies

All experimental procedures were performed in accordance with protocols approved by the Institutional Laboratory Animal Care and Use Committee of Zhengzhou University. All animals received humane care according to the criteria outlined in the “Guide for the Care and Use of Laboratory Animals Chinese Version” (2006). SJSA-1 cells (5 million cells per tumor) suspended in 0.1 mL of Matrigel were implanted subcutaneously into the flank of 5-week-old athymic nude mice (Hunan Slack King of laboratory animals, Changsha, China). Once tumors reached a volume of at least 120 mm^3^, the mice were randomly assigned to the control or treated groups (8 mice per group). Tumor-bearing mice were treated twice (BID) per day with 30 mg/kg MLN4924 or an equal amount vehicle as a control for 2 weeks. The tumor volumes were recorded using Vernier calipers every two days and calculated by the following equation: V = ab^2^/2, where ‘a’ represents the length and ‘b’ represents the width, and then transformed into relative values (V) using the formula: V = Vt/V0, where V0 is the initial tumor volume and Vt is the final tumor volume after sacrifice. To investigate the mechanism of MLN4924-induced anti-tumor activity, tumor tissues were harvested from the mice after sacrifice and either fixed in formalin for IHC staining of p21 and p27 or used for the H&E staining assay.

### Statistical analyses

All data are displayed as the mean ± SE unless otherwise specified. The methods used to evaluate statistical significance (*p* < 0.05 was deemed significant) are indicated in the text and/or figure legends.

## SUPPLEMENTARY FIGURES


