# Supplementary material for: MLN4924 suppresses neddylation and induces cell cycle arrest, senescence, and apoptosis in human osteosarcoma
Source: Oncotarget. 2016 May 19;7(29):45263–74. doi: 10.18632/oncotarget.9481 (PMC5216721; doi:10.18632/oncotarget.9481)
Supplement: Supplementary file 1 [file oncotarget-07-45263-s001.pdf]

MLN4924 suppresses neddylation and induces cell cycle arrest, senescence, and apoptosis in human osteosarcoma

SUPPLEMENTARY FIGURES

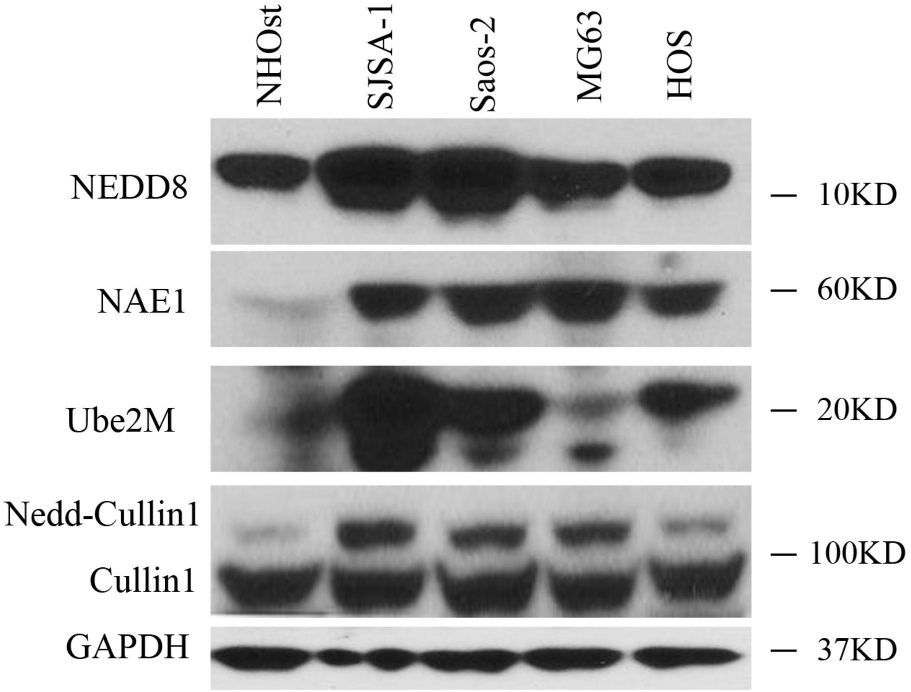

Supplementary Figure S1: The levels of key components of neddylation pathway in osteosarcoma and normal Human Osteoblasts (NHOst) cells. The levels of NEDD8, NAE1, Ube2M, Cullin1 (including Nedd-cullin1) in osteosarcoma cell lines and in normal human osteoblasts (NHOst) were examined by western blotting analysis. GAPDH was used as a loading control.

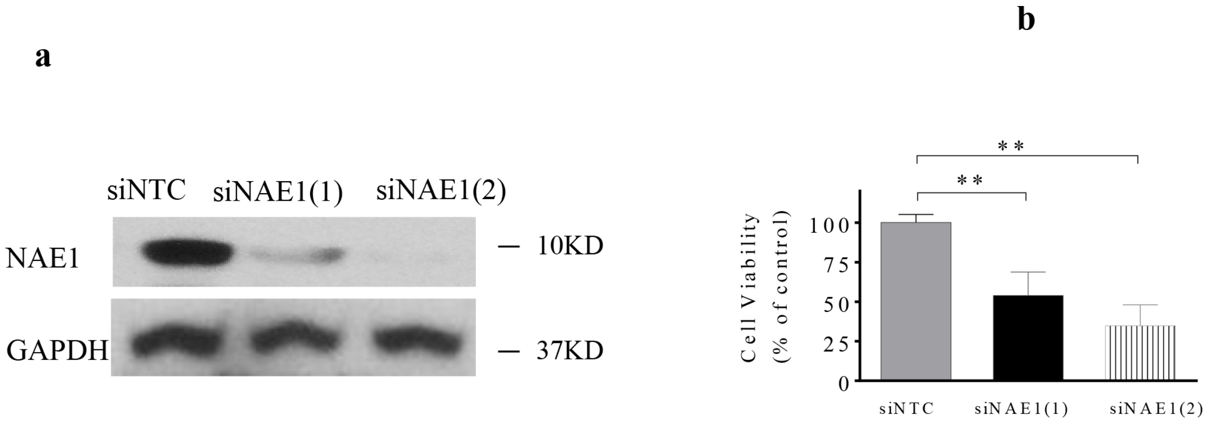

Supplementary Figure S2: Knockdown of NAE1 significantly inhibits cell viability of SJSA-1 cell line. a. SJSA-1 cell line was transfected with control siRNA (siNTC), siRNA against NAE1 (siNAE1 (1) and siNAE1 (2)) for 48 h. The level of NAE1 was examined by western blotting analysis. GAPDH was used as a loading control. **b.** Cell viability was determined in cells transfected with siRNA for 4 days with trypan blue assay. Two siNAE1 oligos against were purchased from GE Dharmacon, Shanghai, China.

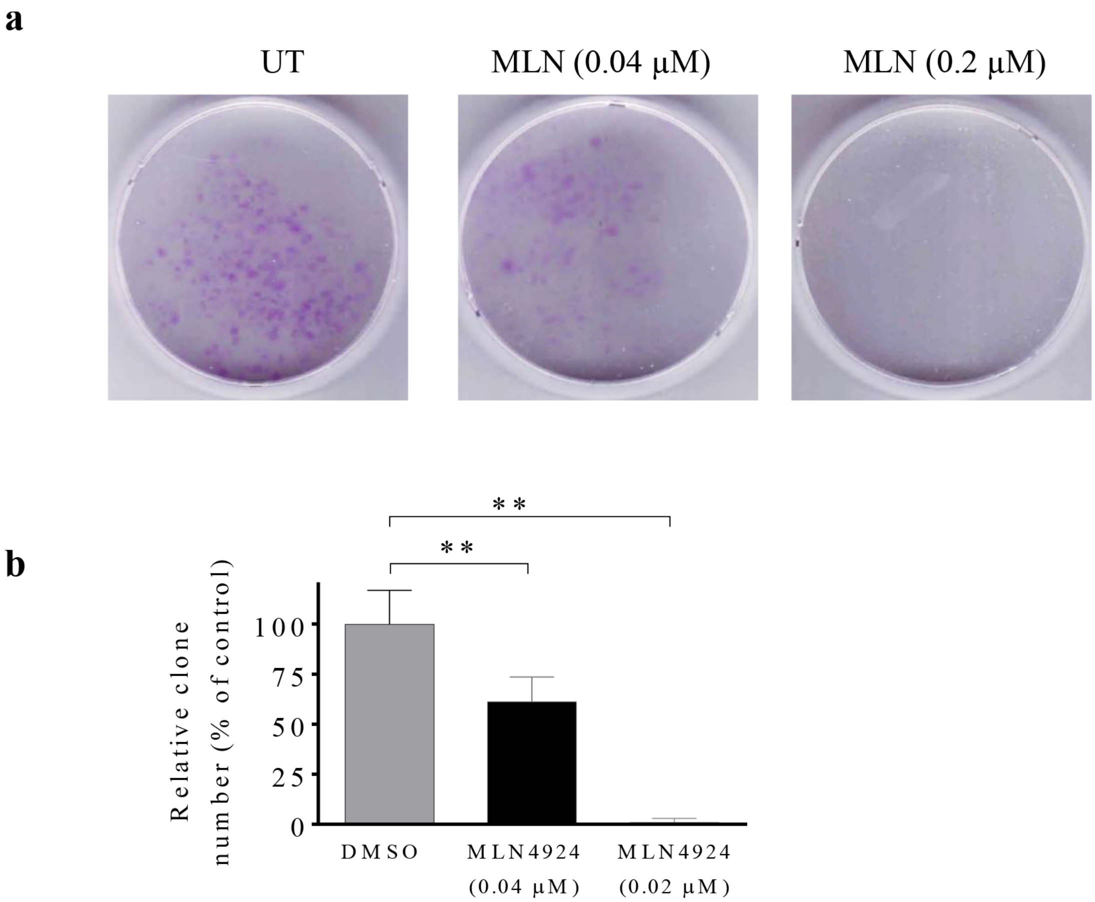

Supplementary Figure S3: MLN4924 inhibits clonogenic formation in SJSA-1 cell line. a. Human OS SJSA-1 cell line was seeded into 60 mm × 15 mm petri-dish at 3,000 cells per well in triplicates and treated with MLN4924 for 12 days, followed by 0.01% (w/v) crystal violet staining and colony counting. Representative images of three independent experiments are shown for colony formation. **b.** graph of the relative number of colonies formed (the result of three independent experiments, expressed as mean ± SEM). (***p*<0.01).

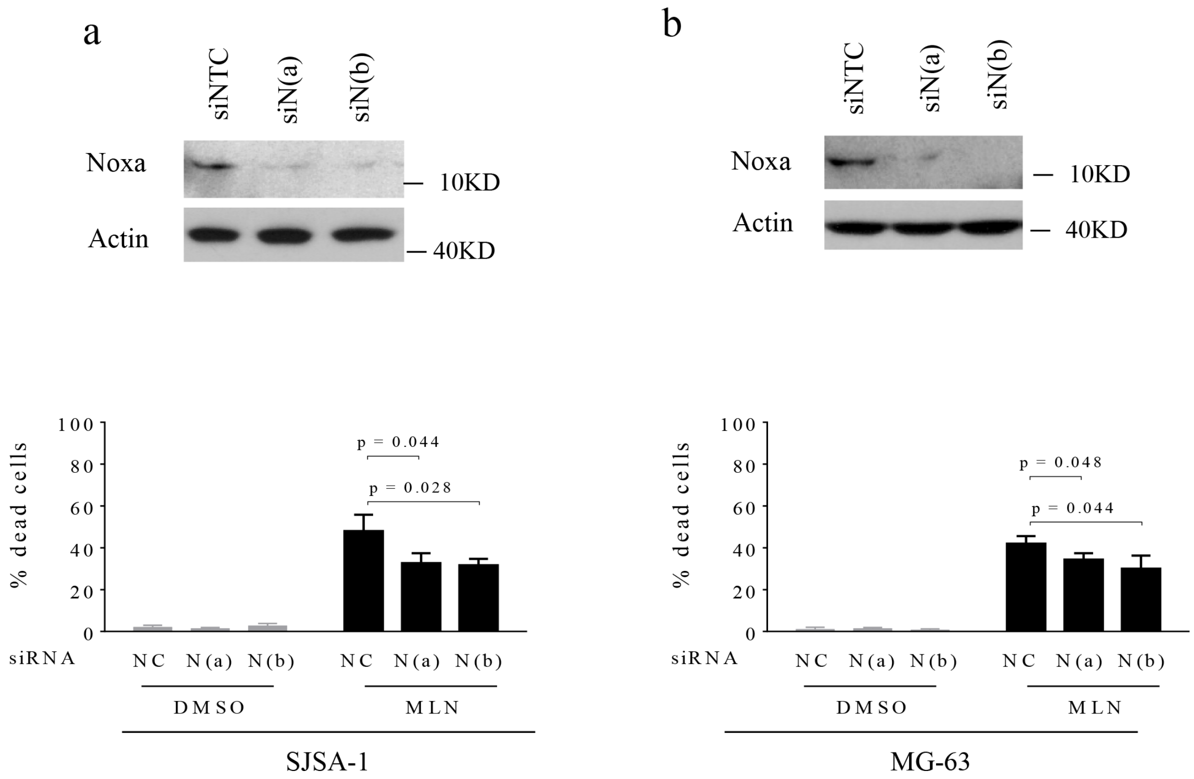

Supplementary Figure S4: Noxa plays a role in MLN4924-mediated anticancer activity in OS cells. a. SJSA-1 and MG-63 cell lines transfected with siRNA oligos against non-targeting control (siNTC) or Noxa (siN(a) and siN(b)) were treated with 1 μM of MLN4924 for 48 h. The transfection efficacy was examined by western blotting analysis. Actin was used as a loading control. Transfected cells were treated for another 48 h, cell death was examined by trypan blue staining and microscopic counting.
